# Long-term physical activity, brain structure, and cognitive function: a systematic review of longitudinal observational evidence

**DOI:** 10.1186/s12883-026-05055-5

**Published:** 2026-06-11

**Authors:** Dilip Shettigar, Suresh Sukumar, Rajagopal Kadavigere, K Vaishali, Nitika C Panakkal, Winniecia Dkhar, Abhimanyu Pradhan, Baskaran Chandrasekaran, Hari Prakash Palaniswamy, Poovitha Shruthi Paramashiva, Sneha Ravichandran, Sathya Sabina Muthu, Koustubh Kamath

**Affiliations:** 1 Department of Medical Imaging Technology, Manipal College of Health Professions, Manipal Academy of Higher Education, Manipal, India; 2 Department of Radiodiagnosis and Imaging, Kasturba Medical College, Manipal Academy of Higher Education, Manipal , India; 3 Department of Physiotherapy, Manipal College of Health Professions, Manipal Academy of Higher Education, Manipal, India; 4 Symbiosis School Of Sports Sciences, Symbiosis International (Deemed University), Pune, India; 5 Department of Speech and Hearing, Manipal College of Health Professions, Manipal Academy of Higher Education, Manipal, India; 6 Division of Yoga, Centre for Integrative Medicine and Research, Manipal Academy of Higher Education, Manipal, India

**Keywords:** Physical activity, Brain volume, White matter integrity, Cognitive function, Hippocampus, Executive function, Longitudinal cohort, Neuroimaging, Dementia, Ageing

## Abstract

**Background:**

Physical inactivity is a key modifiable risk factor for dementia, and physical activity is proposed to confer neuroprotective effects on brain structure and cognitive function.

**Objectives:**

To synthesize longitudinal observational evidence on associations between long-term habitual physical activity and brain structural outcomes and cognitive function across adult age groups.

**Methods:**

Five databases were searched from inception to August 2024. Eligible studies enrolled adults aged ≥ 18 years without cognitive impairment or dementia, used validated accelerometry or self-report physical activity assessment, and employed longitudinal observational designs with a minimum 12-month exposure-to-outcome window. Risk of bias was assessed using the ROBINS-E tool.

**Results:**

Twenty studies were included in the review. Higher habitual physical activity was consistently associated with preserved hippocampal and prefrontal grey matter volume and attenuated white matter microstructural decline. Executive function and processing speed showed the most consistent cognitive associations, with evidence of a dose-response accumulation effect across adulthood. Associations appeared amplified in APOE-ε4 carriers. Bidirectional analyses indicated preserved brain structure also predicted subsequent physical activity, suggesting reverse causation cannot be excluded. All 20 studies were rated “some concerns” on the ROBINS-E scale.

**Conclusion:**

Longitudinal observational evidence is consistent with the hypothesis that sustained habitual physical activity is associated with preserved brain structure and attenuated cognitive decline, though causal inference is not warranted given the limitations of the observational design.

**Supplementary Information:**

The online version contains supplementary material available at 10.1186/s12883-026-05055-5.

## Introduction

Cognitive decline and dementia represent one of the most pressing global public health challenges of the twenty-first century. According to the Global Burden of Disease Study 2019, approximately 57.4 million individuals were living with dementia worldwide, with projections estimating this number will nearly triple to 152.8 million by 2050, driven primarily by population aging and growth [1]. The 2024 Lancet Commission on Dementia Prevention, Intervention, and Care identified 14 modifiable risk factors, including physical inactivity, that collectively account for approximately 45% of all global dementia cases [2]. These figures underscore an urgent imperative to identify cost-effective, accessible, and scalable strategies to preserve brain health across all age groups.

Physical activity has emerged as one of the most promising non-pharmacological interventions for promoting brain health. Epidemiological evidence consistently demonstrates that higher levels of physical activity are associated with a reduced risk of cognitive decline, dementia, and Alzheimer’s disease [3, 4]. Unlike pharmacological agents, regular physical activity is widely accessible, has minimal adverse effects, and confers well-established cardiovascular and metabolic benefits, in addition to its neurocognitive properties [5]. A study demonstrated that twelve months of aerobic exercise training increased anterior hippocampal volume by approximately 2% in older adults, effectively reversing one to two years of age-related volume loss [6]. This hippocampal expansion was accompanied by significant improvements in spatial memory and elevated serum levels of brain-derived neurotrophic factor (BDNF), implicating dentate gyrus neurogenesis as a key mechanism [6]. Cross-sectional studies have further confirmed that the duration and frequency of weekly exercise positively correlate with hippocampal grey matter volume in healthy adults across a broad age range [7]. A systematic review concludes that physical activity effects on brain structure are regionally specific to areas vulnerable to dementia, and that protective effects appear greater in individuals at genetic risk [8].

Beyond grey matter volumetry, the integrity of cerebral white matter is another important index of brain structural health. White matter consists primarily of myelinated neural fibers connecting distributed cortical and subcortical regions, and its degradation with advancing age is associated with slowed information processing, reduced network connectivity, and elevated risk of neurological disorders [9]. Studies compiling the evidence from structured exercise training interventions have demonstrated that aerobic, resistance, and concurrent training programs can produce measurable changes in grey matter volume, white matter integrity, and functional brain connectivity in older adults [9–11]. This intervention literature establishes that the brain is structurally responsive to exercise, but the controlled and relatively short-duration nature of these trials limits their ecological validity for understanding the sustained neurological impact of lifelong physical activity patterns.

Parallel to these structural brain changes, studies have documented beneficial effects of sustained physical activity on multiple cognitive domains, including executive function, processing speed, attention, and episodic memory [3, 4, 12]. A meta-analysis examining the association between physical activity and risk of cognitive decline in non-demented adults provides foundational evidence that habitual physical activity engagement in everyday life is prospectively associated with reduced cognitive decline [13].

Despite the burgeoning literature, several important gaps remain. Existing systematic reviews have often focused on short-term exercise interventions or single cognitive or structural outcomes, without integrating both neuroimaging and neuropsychological data within a unified framework [8, 9]. The differential effects of long-term physical activity on region-specific brain volumes and on specific cognitive domains across different age strata remain insufficiently characterized. The exclusive inclusion of longitudinal observational designs in the present review is necessary to capture the cumulative, sustained effects of habitual physical activity in real-world settings, distinct from the constrained environments of short-duration exercise trials. To address these gaps, this systematic review aims to synthesize evidence from longitudinal observational studies to determine how long-term changes in physical activity levels affect brain structure and cognitive function across different adult age groups. Specifically, the review will examine associations among habitual physical activity, brain volume, white matter integrity, and performance on neuropsychological assessments of cognitive function.

## Methods

This systematic review was designed and conducted in accordance with the Preferred Reporting Items for Systematic Reviews and Meta-Analyses (PRISMA) 2020 guidelines [14]. The review protocol was prospectively registered in the PROSPERO International Prospective Register of Systematic Reviews (Registration ID: CRD42024595135).

### Eligibility criteria

Studies were selected for inclusion based on the Population, Exposure, Comparator, Outcome, and Study Design (PECOS) framework Table 1.

**Table 1:** Eligibility criteria

Population	Studies were eligible for inclusion if they enrolled adults > 18 years of age, regardless of sex, ethnicity, or geographic region. Studies that included individuals with a pre-existing diagnosis of cognitive impairment, dementia, or Alzheimer’s disease were excluded, as the primary focus of this review is the trajectory of brain structural and cognitive changes in non-demented adults.
Exposure	The exposure of interest was long-term physical activity, defined as any structured or habitual engagement in aerobic exercise, resistance training, or combined exercise modalities, with a minimum of 12 months between the physical activity exposure assessment and the primary outcome measurement. This threshold was applied to distinguish sustained habitual physical activity patterns from the effects of short-term or acute exercise, and to ensure that observed associations reflected long-term behavioural engagement rather than transient physiological responses. Physical activity was required to have been assessed using validated objective or self-report instruments, including accelerometry or validated self-report questionnaires capturing frequency, duration, and/or intensity of physical activity engagement. Studies in which the primary exposure was cardiorespiratory fitness measured via graded exercise testing, or any other physiological performance parameter not directly reflecting behavioural physical activity, were excluded.
Comparator	Studies were required to include a comparator condition, defined as either a sedentary or low-activity group, or baseline measurements within the same cohort, enabling the assessment of longitudinal change in brain structure and cognitive function relative to physical activity levels.
Outcomes	The primary outcomes of interest were: (i) brain volume, including total brain volume, regional grey matter volume (with particular emphasis on hippocampal, prefrontal, and temporal regions), and white matter volume; and (ii) white matter integrity, as assessed by diffusion tensor imaging (DTI) metrics including fractional anisotropy (FA) and mean diffusivity (MD). Secondary outcomes included performance on standardised neuropsychological assessments of cognitive function, encompassing executive function, processing speed, episodic memory, and attention.
Study design	Only longitudinal observational studies including prospective cohort studies, retrospective cohort studies, and longitudinal panel studies were eligible for inclusion. Randomised controlled trials (RCTs), cross-sectional studies, case-control studies, case reports, editorials, opinion articles were excluded.

### Information sources

A comprehensive and systematic search of five electronic bibliographic databases was conducted: PubMed, Web of Science, Scopus, OVID, and Embase. The search was conducted from the inception of each database up to August 2024. No language restrictions were applied at the search stage; however, studies published in languages other than English were excluded if no validated translation was available.

### Search strategy

The search strategy was developed in consultation with a librarian and employed a combination of Medical Subject Headings (MeSH) terms and free-text keywords relating to three conceptual domains: (i) physical activity and exercise; (ii) brain structure and neuroimaging; and (iii) cognitive function. Boolean operators (AND, OR) were used to combine terms within and across domains. The full search strategy for each database is provided in the supplementary file. In addition to database searches, the reference lists of all included studies and relevant systematic reviews were hand-searched to identify any additional eligible studies not captured by the electronic search.

### Selection process

All records identified through database searches were imported into Rayyan, a systematic review management software [15], and duplicates were removed before screening. Study selection was performed in two sequential stages by two independent review authors (DS and SS).

In the first stage, titles and abstracts of all retrieved records were screened against the pre-specified eligibility criteria. Records that were clearly irrelevant were excluded at this stage. In the second stage, the full texts of all potentially eligible articles were retrieved and assessed in detail against the eligibility criteria. Any disagreements between the two reviewers at either stage were resolved through discussion and consensus; where consensus could not be reached, a third reviewer (RK) served as the arbiter. The reasons for excluding full-text articles were documented and reported in the PRISMA flow diagram.

### Data collection process and data items

Data from all included studies were extracted independently by two review authors (DS and SR) using a standardised, pre-piloted data extraction form developed a priori. Discrepancies were resolved by consensus or adjudication by the third reviewer (SM). The information extracted from each included study is shown in Table 2.

**Table 2:** Data extraction elements

Study characteristics	First author, year of publication, country, study design, follow-up duration, and sample size.
Population characteristics	Age range, mean age, sex distribution, and relevant baseline clinical characteristics.
Exposure details	type, frequency, duration, and intensity of physical activity; method of assessment; and definition of active versus inactive groups.
Neuroimaging outcomes	MRI acquisition parameters, brain regions assessed, volumetric measures, and DTI metrics (FA, MD).
Cognitive outcomes	neuropsychological tests administered, cognitive domains assessed, and reported summary statistics.
Confounders and covariates	Variables adjusted for in multivariate analyses.

### Study risk of bias assessment

The methodological quality and risk of bias of all included studies were assessed independently by two review authors (NP and KV) using the Risk Of Bias In Non-randomized Studies of Exposures (ROBINS-E) tool [16]. The ROBINS-E tool is specifically designed for the assessment of observational studies examining the effect of exposures as distinct from interventions and evaluates risk of bias across seven domains: (i) bias due to confounding; (ii) bias arising from measurement of the exposure; (iii) bias in selection of participants into the study; (iv) bias due to post-exposure interventions; (v) bias due to missing data; (vi) bias in measurement of the outcome; and (vii) bias in selection of the reported result. Each domain was rated as presenting a low, some concerns, high, or very high risk of bias, and an overall risk of bias judgment was assigned to each study based on the most critical domain-level rating. Disagreements between the two reviewers were resolved through discussion and consensus; where agreement could not be reached, the third reviewer (KK) served as the arbiter.

### Synthesis methods

Given the anticipated heterogeneity across included studies in population characteristics, physical activity exposure definitions, neuroimaging acquisition protocols, and outcome measurement instruments, a narrative synthesis was planned a priori as the primary method of evidence synthesis. The narrative synthesis was conducted in accordance with the Synthesis Without Meta-analysis (SWiM) reporting guideline [17] to ensure transparency and reproducibility of the synthesis process.

The findings of all included studies were synthesized narratively and structured according to the primary outcomes (brain volume and white matter integrity) and secondary outcomes (cognitive function), with further sub-grouping by age category (young adults, middle-aged adults, and older adults) and brain region of interest. For each outcome, the direction, magnitude, and consistency of associations between long-term physical activity and the respective brain structural or cognitive measure were described and tabulated across studies. Where studies reported effect estimates, these were extracted and presented alongside their associated measures of uncertainty to facilitate qualitative comparison. Similarities and differences in findings across studies were explored in relation to variations in study population, follow-up duration, type and intensity of physical activity exposure, and outcome assessment methodology.

## Results

### Study selection

**Fig. 1:**
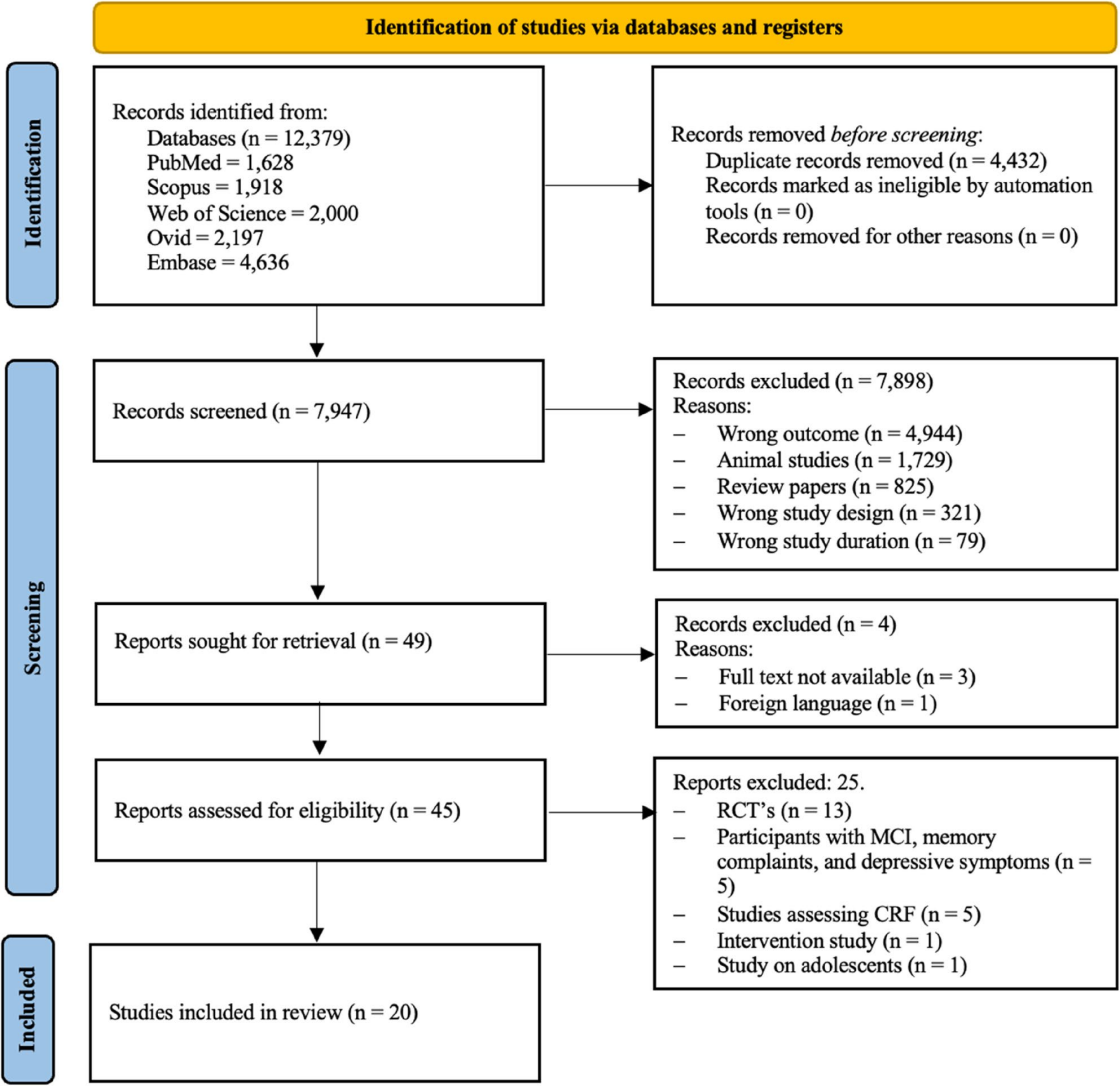
Study selection flow chart

The electronic database search returned 12,379 records. Following the removal of 4,432 duplicate records, 7,947 records were screened at the title and abstract stage. Of these, 7,898 records were excluded for the following reasons. Forty-nine records were sought for full-text retrieval, of which four could not be obtained. Following full-text review, 25 reports were excluded. A total of 20 studies were included in the final review. The complete study selection process is depicted in the PRISMA flow diagram (Fig. 1).

### Study characteristics

All 20 included studies employed prospective longitudinal study designs. Follow-up durations ranged from 18 months to 30 years. The majority of studies (55%) were conducted in the United States [18–28], with additional studies from Iceland [29], Australia [30], the Netherlands [31], Switzerland [32], Canada [33], the United Kingdom [34–36], and a multicenter European cohort [37]. Several studies utilized large, well-established population-based cohort platforms, including the UK Biobank [34, 36], the Baltimore Longitudinal Study of Aging (BLSA) [26], the Health, Aging and Body Composition (Health ABC) Study [18–20], the Coronary Artery Risk Development in Young Adults (CARDIA) Study [21, 27], and the Atherosclerosis Risk in Communities (ARIC) Study [23] (Table 3).

**Table 3:** Study and participant characteristics

Author, Country/Cohort	Study design	Study duration	Sample size	Baseline age	Sex distribution	Population type
Ai et al., 2024 [32] Canada / PREVENT-AD	PLC	4 years	156	66.07 ± 4.88 years	42 males	Older adults at high risk of Alzheimer’s disease
Arnardottir et al., 2016 [28] Iceland / AGES-Reykjavik Study	PLC	5 years	352	79.1 ± 4.4 years	137 males	Community-dwelling older adults
Barha et al., 2020 [17] USA / Health, Aging and Body Composition (Health ABC) Study	PLC	10 years	*n* = 2,873 for longitudinal cognition analysis; MRI subsample *n* = 303	70–79 years	1376 males in cognition analysis; 126 males in MRI subsample	Community-dwelling older adults
Best et al., 2015 [19] USA / Health ABC Study (*conference abstract*)	PLC	10-year PA exposure + 3-year MRI follow-up (Year 10–13)	313	Mean 72.9 years at Year 1	Not reported	Community-dwelling older adults
Best et al., 2017 [18] USA / Health ABC Study	PLC	10-year PA exposure + 3-year MRI follow-up (Year 10–13)	141	Mean age 72.5 years at baseline; ~82.5 years at first MRI	60% female	Community-dwelling older adults
Fraser et al., 2022 [29] Australia / PATH Through Life Project	PLC	12 years follow-up (multiple MRI waves every ~ 4 years)	*n* = 786 participants (411 middle-aged; 375 older adults)	Middle-aged: mean 47.2 years; Older adults: mean 63.1 years	Middle-aged: 44.5% male; Older adults: 52% male	Community-dwelling healthy adults without dementia, stroke, Parkinson’s disease, or epilepsy
Frederiksen et al., 2015 [36] Multicenter European cohort / Leukoaraiosis and Disability Study (LADIS)	PLC	3 years	282	73.1 ± 5.1 years	164 females	Non-demented elderly with age-related white matter changes (ARWMC)
Gafni et al., 2022 [20] USA / Coronary Artery Risk Development in Young Adults (CARDIA) Study	PLC	30-year cohort, with 20-year physical activity trajectories and cognition assessed at year 30	1,939	25.2 ± 3.5 years	58% female	Community-based cohort of young adults
Greendale et al., 2021 [21] USA / Study of Women’s Health Across the Nation (SWAN)	PLC	Median follow-up 11.9 years	1,718	45.7 ± 2.5 years	100% female	Community-dwelling midlife women from a multi-ethnic US cohort
Hofman et al., 2023 [30] Netherlands / Rotterdam Study	PLC	Median follow-up ≈ 5 years	4,365	64.0 ± 10.8 years	56% female	Community-dwelling middle-aged and older adults
Hotz et al.2023 [31] Switzerland / Longitudinal Healthy Aging Brain (LHAB) Study	PLC	7-years (baseline, 1-year, 2-year, 4-year, 7-year assessments)	231	70.8 ± 5.1 years	48.9% female	Community-dwelling cognitively healthy older adults
Huang et al.2022 [33] United Kingdom / UK Biobank cohort	PLC	Median follow-up: 9.04 years	431,924	Median age 58 years	46% male	General population middle-aged adults
James et al.2023 [34] United Kingdom / 1946 British Birth Cohort	PLC	30-year follow-up (physical activity measured at ages 36, 43, 53, 60–64, and 69 years)	1,417	36 to 69 years	53% female	Community-dwelling adults from a nationally representative birth cohort
Palta et al.2021 [22] USA / Atherosclerosis Risk in Communities (ARIC) Study / ARIC Neurocognitive Study	PLC	Approximately 25 years follow-up	1,604	53 years at baseline, 76.2 ± 5.3 years at MRI	61% female	Community-dwelling middle-aged adults
Reas et al.2019 [23] USA / Rancho Bernardo Study of Healthy Aging	PLC	27 years with repeated cognitive assessments every ~ 4 years	2,027	73.5 ± 9.0 years	60% female	Community-dwelling older adults
Rodriguez-Ayllon et al.2024 [35] United Kingdom / UK Biobank cohort	PLC	~ 2.1 years between two MRI visits	3,027	62.45 ± 7.27 years	51.3% female	Community-dwelling middle-aged and older adults
Smith et al.2014 [24] USA	PLC	18 months	97	65–89 years	69 females	Cognitively healthy older adults stratified by APOE-ε4 genetic risk
Tian et al.2022 [25] USA / Baltimore Longitudinal Study of Aging (BLSA)	PLC	3.8 ± 2.4 years	248	71.3 ± 8.4 years	51% female	Community-dwelling older adults
Whitaker et al.2021 [26] USA / Coronary Artery Risk Development in Young Adults (CARDIA) Study	PLC	10 years	1,970	38–50 years	58.3% female	Community-based Black and White middle-aged adults
Willey et al.2016 [27] USA / Northern Manhattan Study (NOMAS)	PLC	5 years	Baseline = 1,228; Follow-up = 876	70.6 ± 9.0 years	60.9% female	Community-dwelling racially/ethnically diverse older adults

Abbreviations: *PLC *Prospective longitudinal cohort

### Population characteristics

Sample sizes varied considerably across studies, ranging from 97 to 431,924 participants. The majority of studies enrolled community-dwelling older adults (aged ≥ 60 years), while several focused on middle-aged adults [22, 23, 27] or younger adults [21]. One study enrolled populations enriched for Alzheimer’s disease risk, defined by family history [33]. The sex distribution was broadly balanced across studies, though one study exclusively enrolled women [22]. Baseline ages ranged from approximately 25.2 years to 79.1 years (Table 3).

### Physical activity exposure assessment

Physical activity was assessed using various methods across the 20 included studies (Table 4). Self-reported measures were the most commonly used, encompassing a range of validated instruments including the Minnesota Leisure-Time Physical Activity Questionnaire [35], the Kaiser Physical Activity Survey (KPAS) [22], the Modified Baecke Physical Activity Questionnaire [23], the Stanford Brief Activity Survey (SBAS) [25], the Physical Activity History questionnaire (PAH) [21], the Lifetime Total Physical Activity Questionnaire [33], the LASA Physical Activity Questionnaire (LAPAQ) [31], and the modified International Physical Activity Questionnaire (IPAQ) [34]. Objective assessment of physical activity was employed in three studies [26, 27, 29]. Arnardottir et al. [29] quantified total physical activity counts per day using a hip-worn ActiGraph GT3X accelerometer worn over seven consecutive days; Tian et al. (2022) measured relative moderate-to-vigorous physical activity (MVPA) using an Actiheart accelerometer, with intensity defined by heart rate reserve (≥ 60% HRR); and Whitaker et al. [27] measured sedentary behaviour, light physical activity (LPA), and MVPA using an ActiGraph 7164 accelerometer, with intensity thresholds defined by Freedson cut points (≥ 1952 counts/min). The dominant physical activity types assessed included MVPA, leisure-time physical activity (LTPA), total physical activity, and domain-specific activities such as walking, sport, and household activities.

**Table 4:** Physical activity exposure

Author	PA type	PA measurement	PA duration	PA intensity
Ai et al.(Ai et al., 2024) [32]	Self-reported	Lifetime Total Physical Activity Questionnaire	Self-reported PA during midlife (~ 40 years) and later life (previous year)	Light, moderate, vigorous
Arnardottir et al.(Arnardottir et al., 2016) [28]	Objective measure	Hip-worn ActiGraph GT3X accelerometer	7 consecutive days	Total PA counts/day; lifestyle PA ≥ 760 counts/min; sedentary behaviour defined as < 100 counts/min
Barha et al.(Barha et al., 2020) [17]	Self-reported	Walking minutes/week using a standardized Health ABC questionnaire	Longitudinal walking behavior measured annually for 10 years	NR
Best et al.(Best et al., 2015) [19] (*conference abstract*)	Walking	Self-reported walking minutes/week collected annually	10-year physical activity trajectory (Year 1–10)	Not reported
Best et al.(Best et al., 2017) [18]	Walking	Self-reported walking minutes/week collected annually	10-year physical activity trajectory (Year 1–10)	Moderate-intensity walking
Fraser et al.(Fraser et al., 2022) [29]	Total physical activity (mild, moderate, vigorous activities)	Self-reported physical activity recall questionnaire	Long-term physical activity change measured across multiple waves over up to 12 years	MET-based intensity categories: mild (~ 3 METs), moderate (~ 6 METs), vigorous (~ 9 METs)
Frederiksen et al.(Frederiksen et al., 2015) [36]	General physical activity (lifestyle activity)	Self-reported physical activity classified as active (> 30 min activity ≥ 3 days/week) or inactive (< 30 min activity ≥ 3 days/week)	Baseline PA with 3-year cognitive follow-up	Moderate habitual activity
Gafni et al.(Gafni et al., 2022) [20]	MVPA	Physical Activity History questionnaire (PAH)	Repeated PA measurements across 20 years	MVPA
Greendale et al.(Greendale et al., 2021) [21]	Sport and exercise physical activity	Kaiser Physical Activity Survey (KPAS)	Repeated PA assessments across ~ 12 years	Self-reported sport/exercise activities with perceived exertion levels
Hofman et al.(Hofman et al., 2023) [30]	Total physical activity and domain-specific activity (walking, cycling, sports, gardening, domestic work)	LASA Physical Activity Questionnaire (LAPAQ)	Physical activity assessed over previous 2 weeks, repeated at two study waves	MET-based intensity across activities
Hotz et al.(Hotz et al., 2023) [31]	Leisure physical activity	Expanded Victoria Longitudinal Study Activity Questionnaire	Leisure activity assessed across five time points over 7 years	Frequency-based leisure activity scores
Huang et al.(Huang et al., 2022) [33]	Total physical activity and leisure-time physical activity (LTPA)	Modified International Physical Activity Questionnaire (IPAQ)	Based on typical activity during the past 4 weeks	MET-based categories: walking (3.3 METs), moderate (4 METs), vigorous (8 METs); LTPA categorized into low (< 400), moderate (400–1200), high (≥ 1200 MET-min/week)
James et al.(James et al., 2023) [34]	Leisure-time physical activity	Minnesota Leisure-Time Physical Activity Questionnaire and EPIC-PAQ-2	Frequency categories per month across adulthood	Categorized as: • Inactive: no activity/month • Moderately active: 1–4 times/month • Most active: ≥5 times/month
Palta et al.(Palta et al., 2021) [22]	Leisure-time physical activity (LTPA)	Modified Baecke Physical Activity Questionnaire	PA measured at midlife (1987–1989) and late life (2011–2013)	Categorized by moderate-to-vigorous physical activity (MVPA): none (0 min/week), low (1–74), middle (75–149), high (≥ 150 min/week)
Reas et al.(Reas et al., 2019) [23]	Physical activity across life stages: teenage years, age 30, and older age exercise	Self-reported questionnaire	Lifetime PA assessment; concurrent PA measured at each cognitive assessment over 27 years	NR
Rodriguez-Ayllon et al.(Rodriguez-Ayllon et al., 2024) [35]	Walking, strenuous sports, household activities, and total physical activity	Self-reported leisure-time physical activity questionnaire	Physical activity assessed at two imaging visits (~ 2 years apart)	Activity types categorized by domain (walking, strenuous sports, household activities) rather than MET-based intensity
Smith et al.(Smith et al., 2014) [24]	Leisure-time physical activity	Stanford Brief Activity Survey (SBAS)	Habitual physical activity (classified as High PA vs. Low PA)	Moderate-to-vigorous physical activity ≥ 3 days/week vs. low intensity ≤ 2 days/week
Tian et al.(Tian et al., 2022) [25]	Moderate-to-vigorous physical activity (MVPA)	Actiheart accelerometer	15 ± 22 min/day of relative MVPA	Relative intensity defined using heart rate reserve (≥ 60% HRR); absolute intensity defined using population activity count thresholds
Whitaker et al.(Whitaker et al., 2021) [26]	Sedentary behavior (SED), light physical activity (LPA), and moderate-to-vigorous physical activity (MVPA)	ActiGraph 7164 accelerometer	Accelerometer worn ≥ 4 days, ≥ 10 h/day	Defined using Freedson cut points: SED ≤ 100 counts/min; LPA 101–1951 counts/min; MVPA ≥ 1952 counts/min
Willey et al.(Willey et al., 2016) [27]	Leisure-time physical activity (LTPA)	Validated questionnaire adapted from the National Health Interview Survey	Based on reported activity frequency and duration over previous 2 weeks	Classified using MET values: moderate–heavy (≥ 6 METs) vs. no/light activity

### Brain structural outcomes

#### Grey matter

Ten studies examined associations between long-term physical activity and grey matter brain volumes using structural MRI (Table 5). The most consistently studied region was the hippocampus, with findings broadly supporting a positive association between greater physical activity and preserved hippocampal volume.

**Table 5:** Brain imaging and cognitive outcomes

Author	Brain regions	Cognitive domains	Cognitive tests	Confounders	Main findings
Ai et al.(Ai et al., 2024) [32]	Prefrontal cortex, hippocampus, supplementary motor area, cingulate cortex, cerebellum	Immediate memory, visuospatial ability, language, attention, delayed memory	Repeatable Battery for the Assessment of Neuropsychological Status (RBANS)	Age, sex, education, APOE-ε4 status, later-life physical activity, intracranial volume, head motion	Midlife MVPA was associated with greater surface area of the left middle frontal gyrus (DLPFC) (cluster = 376.12 mm²; vertex-wise *p* < 0.001, cluster-wise *p* < 0.05). Midlife total PA was associated with altered resting-state connectivity between the middle frontal gyrus and supplementary motor area/cingulate gyrus and increased connectivity between hippocampus and cerebellum (F = 7.1, voxel *p* < 0.001, cluster pFWE < 0.05). No significant association was observed between midlife PA and longitudinal cognitive change over 4 years.
Arnardottir et al.(Arnardottir et al., 2016) [28]	Global gray matter (GM) and white matter (WM) volumes	NA	MMSE and DSST used only for participant screening	Age, sex, education, brain infarcts, time between MRI scans, baseline self-reported PA, BMI, depression, mean arterial pressure, type 2 diabetes, smoking status, lifestyle PA and accelerometer wear time	Higher objectively measured physical activity was associated with greater baseline GM (β = 0.11, *p* = 0.044) and WM volume (β = 0.11, *p* = 0.030) and with less 5-year GM (β = 0.14, *p* = 0.0037) and WM decline (β = 0.11, *p* = 0.030). Sedentary behavior was associated with greater 5-year WM atrophy (β = −0.11, *p* = 0.0007)
Barha et al.(Barha et al., 2020) [17]	Hippocampus and dorsolateral prefrontal cortex (DLPFC)	Global cognition; executive function; processing speed	Modified Mini-Mental State Examination (3MS) and Digit Symbol Substitution Test (DSST)	Age, race, education, study site, BMI, diabetes, cardiovascular disease, cerebrovascular disease, brain atrophy, and total gray matter volume	Maintaining walking over 10 years predicted less decline in executive function and processing speed in females (DSST slope, *p* < 0.01) but not males. Maintenance of walking predicted less decline in global cognition (3MS) in both sexes (*p* = 0.03). Greater maintenance of walking predicted larger left DLPFC volume in females (*p* < 0.01). Walking intercept predicted left hippocampal volume differently by sex (females *p* = 0.03; males *p* = 0.06)
Best et al.(Best et al., 2015) [19] (*conference abstract*)	Global gray matter (GM), white matter hyperintensities (WMH), middle frontal gyrus, anterior cingulate cortex, hippocampus	NA	NA	Age, education, cohort, race, sex, BMI, smoking status, drinking status, diabetes, cardiovascular disease, cerebrovascular disease, gait speed, general cognitive function	Maintenance of walking over 10 years predicted less gray matter volume loss and smaller increases in mean diffusivity from year 10–13 (*p* < 0.05). Higher walking levels were associated with greater middle frontal gyrus and anterior cingulate cortex volume and lower white matter hyperintensity burden, but no association with hippocampal structure.
Best2017(Best et al., 2017) [18]	Hippocampus, global GM, WM, anterior cingulate cortex, dorsolateral prefrontal cortex, and white matter tracts (cingulum, inferior longitudinal fasciculus)	Global cognitive function	3MS	Age, sex, race, education, APOE ε4 status, intracranial volume, BMI, gait speed, chronic diseases (diabetes, stroke, cardiovascular disease), smoking, alcohol use, blood pressure, antihypertensive medication	Better maintenance of walking over 10 years predicted less hippocampal volume loss (*p* = 0.03), smaller increases in gray matter mean diffusivity and white matter axial diffusivity (*p* < 0.01), and better maintenance of global cognitive performance (*p* < 0.01). Individuals maintaining walking showed ~ 4.5% hippocampal volume loss vs. 6.2% in the average participant over 3 years. Changes in white matter axial diffusivity were significantly associated with cognitive changes (β = −0.20, *p* = 0.007).
Fraser et al.(Fraser et al., 2022) [29]	Left and right hippocampal volume	NA	NA	Age, sex, education, BMI, hypertension, diabetes, smoking, depression, baseline physical activity, and APOE-ε4 genotype	In middle-aged adults, each additional 1 MET increase in PA was associated with a 0.03% increase in hippocampal volume (*p* = 0.004). In older adults, PA change was associated with 0.05% larger hippocampal volume per MET increase (*p* = 0.049), but the effect declined by ~ 0.005% per year over time. Effects were stronger among APOE-ε4 carriers, with 0.10% hippocampal volume increase per MET.
Frederiksen et al.(Frederiksen et al., 2015) [36]	White matter changes (ARWMC) assessed on MRI using Fazekas scale	Executive function, processing speed, memory	Trail Making Test A and B, Stroop Test, Verbal Fluency, Symbol Digit Modalities Test, Digit Cancellation, Maze test, and VADAS memory tasks	Age, sex, education, diabetes, stroke history, baseline MMSE score, and white matter lesion severity	Physical activity was associated with better executive function and processing speed at baseline and follow-up. Executive function: baseline β = 0.51, 95% CI 0.13–0.90, *p* = 0.008; follow-up β = 0.24, 95% CI 0.10–0.38, *p* = 0.001. Processing speed: baseline β = 0.48, *p* = 0.005; follow-up β = 0.15, *p* = 0.02. No significant association was found for memory performance.
Gafni et al.(Gafni et al., 2022) [20]	NA	Executive function, processing speed, working memory, verbal memory, and verbal fluency	Stroop Test, Digit Symbol Substitution Test (DSST), Rey Auditory Verbal Learning Test (RAVLT), Montreal Cognitive Assessment (MoCA), Category Fluency, and Letter Fluency	Age, race, sex, smoking, alcohol intake, education, socioeconomic status, and cardiovascular medications	Higher MVPA trajectories over 20 years were associated with better autonomic balance (higher HRV, *p* < 0.001; lower RHR, *p* < 0.0001). HRV mediated the association between PA and category fluency (β ≈ 0.05), but PA was not significantly associated with most cognitive outcomes after multiple comparison correction.
Greendale et al.(Greendale et al., 2021) [21]	NA	Processing speed, verbal episodic memory, working memory	Symbol Digit Modalities Test (SDMT), East Boston Memory Test-Delayed (EBMT-D), Digit Span Backwards (DSB)	Age, race/ethnicity, education, financial hardship, menopause stage, hormone therapy use, depressive symptoms, anxiety, sleep problems, vasomotor symptoms, hypertension, diabetes, and attrition effects	In base models, higher sport/exercise PA was associated with better concurrent cognitive performance: SDMT β = 0.36 (95% CI 0.14–0.59, *p* = 0.002) and EBMT-D β = 0.10 (95% CI 0.05–0.15, *p* < 0.001). PA was also associated with a smaller decline in SDMT (β = 0.06/year, *p* = 0.001). However, after adjusting for demographic factors, menopause-related variables, hypertension, and diabetes, physical activity was not associated with trajectories of any cognitive outcome.
Hofman et al.(Hofman et al., 2023) [30]	Total brain volume, gray matter volume, white matter volume, hippocampal volume, white matter lesion volume	NA	NA	Age, sex, education, intracranial volume, smoking, alcohol intake, BMI, diet quality, hypertension, hypercholesterolemia, diabetes, coronary heart disease, stroke, cancer, and depressive symptoms	No significant association was found between baseline physical activity and later brain structure. However, brain structure predicted future physical activity: larger total brain volume (β = 0.067, pFDR = 0.001), gray matter volume (β = 0.063, pFDR = 0.002), and white matter volume (β = 0.051, pFDR = 0.013) predicted higher sports participation at follow-up. Lower global mean diffusivity predicted higher walking levels (β = −0.074, pFDR = 0.001).
Hotz et al.(Hotz et al., 2023) [31]	Entorhinal cortex thickness (left and right), white matter hyperintensities (WMH), lacunes	Declarative memory	Verbal Learning and Memory Test (VLMT) and Diagnosticum für Cerebralschädigung (DCS) figural memory test	Age, sex, education, antihypertensive medication, obesity, hazardous alcohol consumption, smoking, sleep quality, and leisure activities	Less thinning of the right EC (β = 0.92, *p* < 0.05) and left EC (β = 0.82, *p* < 0.05) was associated with less decline in declarative memory. Thicker baseline EC predicted less memory decline (β = 0.54, *p* < 0.05). Higher baseline physical leisure activity predicted less thinning of the right EC (β = 0.24, *p* < 0.05) and social activity predicted similar protection (β = 0.27, *p* < 0.01). No direct association was observed between WMH/lacunes and declarative memory.
Huang et al.(Huang et al., 2022) [33]	Multiple cortical and subcortical regions including middle temporal gyrus, orbitofrontal cortex, hippocampus, thalamus, inferior parietal cortex, pallidum	NA	Not directly assessed; outcome was incident dementia	Age, sex, education, APOE ε4 status, ethnicity, socioeconomic status, BMI, smoking, alcohol intake, depression, income, employment status, sleep medication, physical impairment, and cerebrovascular disease	Compared with low LTPA, high LTPA reduced dementia risk by 22% (HR = 0.78, 95% CI 0.70–0.93). High sedentary behavior increased dementia risk (HR = 1.22, 95% CI 1.10–1.35). High total physical activity reduced dementia risk (HR = 0.85, 95% CI 0.77–0.93). The lowest dementia risk occurred with 7 h/day sleep, moderate-to-high LTPA, and low-to-moderate sedentary time (HR = 0.59, 95% CI 0.50–0.69). Brain imaging analysis showed associations of PA, sleep, and sedentary behavior with volumes in hippocampus, orbitofrontal cortex, middle temporal gyrus, and thalamus.
James et al.(James et al., 2023) [34]	NA	Cognitive state, episodic verbal memory, processing speed	Addenbrooke’s Cognitive Examination-III (ACE-III), Word Learning Test (WLT), Visual Search Speed (VSS)	Childhood cognition, childhood socioeconomic position, education, cardiovascular health (Framingham risk score), mental health (GHQ-28), APOE-ε4 genotype, smoking, adult socioeconomic position	Being physically active at any adulthood period was associated with higher cognitive performance at age 69. A dose-response accumulation effect was observed: greater number of active periods across adulthood was associated with higher cognitive scores (*p* < 0.01). Individuals active across all five periods had the strongest association with higher ACE-III scores compared with those never active. Associations were attenuated but remained significant after adjustment for childhood cognition, socioeconomic status, and education.
Palta et al.(Palta et al., 2021) [22]	Whole-brain measures including total cortical gray matter, Alzheimer’s disease signature regions, deep gray matter, and white matter structures	NA	NA	Age, sex, education, race-center, APOE-ε4 genotype, smoking status, intracranial volume; sensitivity analyses additionally adjusted for BMI, hypertension, diabetes, and stroke	High midlife MVPA was associated with lower odds of lacunar infarcts in late life (OR = 0.68; 95% CI 0.46–0.99). Higher MVPA was also associated with better white matter integrity (FA difference 0.13 SD, 95% CI 0.004–0.26; MD difference − 0.11 SD, 95% CI − 0.21 to − 0.004). Middle levels of MVPA were associated with greater cortical gray matter volume (β = 0.14 SD, 95% CI 0.06–0.23). Persistently meeting physical activity guidelines in midlife reduced odds of lacunar infarcts (OR = 0.58; 95% CI 0.38–0.90). Overall, greater midlife physical activity was associated with less cerebrovascular brain damage in late life.
Reas et al.(Reas et al., 2019) [23]	NA	Global cognition, executive function, verbal fluency, episodic memory	MMSE, Trail Making Test Part B, Category Fluency Test, Buschke-Fuld Selective Reminding Test	Age, sex, education, birth decade, BMI, smoking status, alcohol consumption, comorbidity index, survival time	Concurrent physical activity showed age-dependent associations with better cognitive performance on all tests: MMSE (*p* = 0.003), Trails B (*p* < 0.001), category fluency (*p* = 0.01), and episodic memory (*p* = 0.02). Physically active individuals showed cognitive performance equivalent to individuals 1–2 years younger at age 70 and ~ 2–2.5 years younger at age 90. Baseline physical activity did not predict rate of cognitive decline over time (*p* > 0.40). Participants active at both age 30 and older age maintained the highest global cognitive function with advancing age (*p* = 0.002).
Rodriguez-Ayllon et al.(Rodriguez-Ayllon et al., 2024) [35]	Total brain volume, gray matter volume, white matter volume, hippocampal volume, frontal cortex volume, and white matter hyperintensities	NA	NA	Age, sex, education, ethnicity, BMI, diet quality, smoking, hypertension, cardiovascular disease, diabetes, depression, and cancer	Greater hippocampal volume at baseline predicted higher physical activity at follow-up (β = 0.048, pFDR = 0.016). Lower white matter hyperintensity burden predicted greater PA (β = −0.040, pFDR = 0.016). Larger hippocampal volume (β = 0.075, pFDR < 0.001), frontal cortex volume (β = 0.043, pFDR = 0.037), and global FA (β = 0.042, pFDR = 0.028) predicted higher household physical activity at follow-up. Conversely, walking was negatively associated with white matter volume over time (β = −0.026, pFDR = 0.008). Overall, evidence suggested that brain structure more strongly predicts future physical activity than physical activity predicting brain structural changes.
Smith et al.(Smith et al., 2014) [24]	Hippocampus, thalamus, caudate, amygdala, caudal middle frontal gyrus, precentral gyrus, total grey matter, cortical white matter	Global cognition, functional status, depressive symptoms	Mini-Mental State Examination (MMSE), Mattis Dementia Rating Scale-2 (DRS-2), Geriatric Depression Scale (GDS), Activities of Daily Living (ADL)	Age, education, sex, APOE genotype, intracranial volume, baseline cognitive performance	A significant interaction between physical activity and genetic risk (APOE-ε4) was observed for hippocampal volume change (*p* = 0.024, partial η² = 0.054). Over 18 months, hippocampal volume decreased by ~ 3% in the High-risk/Low-PA group, while hippocampal volume remained stable in the High-risk/High-PA group and both low-risk groups. No significant PA effects were observed in other brain regions.
Tian et al.(Tian et al., 2022) [25]	Temporal regions (hippocampus, parahippocampal gyrus, entorhinal cortex), white-matter tracts (uncinate fasciculus, hippocampal cingulum), prefrontal cortex, corpus callosum, precentral gyrus, putamen, caudate	NA	NA	Age, sex, race, education, BMI, handedness, APOE-ε4 status, scanner type, gait speed	Longitudinally, higher relative MVPA was associated with slower decline in brain microstructural integrity in temporal regions. Each additional 22 min/day of relative MVPA was associated with less decline in FA of the uncinate fasciculus (β = 0.035, *p* = 0.005) and hippocampal cingulum (β = 0.034, *p* = 0.01) and less increase in MD of the parahippocampal gyrus (β = −0.021, *p* = 0.01) and entorhinal cortex (β = −0.030, *p* = 0.01). No significant associations were observed with absolute MVPA.
Whitaker et al.(Whitaker et al., 2021) [26]	NA	Processing speed, working memory, verbal memory, executive function	Digit Symbol Substitution Test (DSST), Rey Auditory Verbal Learning Test (RAVLT), Stroop Test	Age, sex, race, study center, education, employment status, depressive symptoms, diabetes, hypertension, BMI, smoking, alcohol consumption, sleep quality, snoring	In men, replacing 30 min/day of LPA with MVPA was associated with improved cognition: DSST β = 0.07 (95% CI 0.01–0.14, *p* = 0.019) and RAVLT β = 0.09 (95% CI 0.02–0.17, *p* = 0.009). Replacing SED with MVPA improved RAVLT β = 0.07 (95% CI 0.01–0.13, *p* = 0.037). Replacing SED with LPA was associated with worse DSST β = −0.05 (95% CI − 0.06 to − 0.03, *p* < 0.001) and RAVLT β = −0.03 (95% CI − 0.05 to − 0.01, *p* = 0.014). Associations were largely non-significant in women.
Willey et al.(Willey et al., 2016) [27]	NA	Processing speed, executive function, semantic memory, episodic memory	Color Trails Test, Grooved Pegboard Test, Visual-Motor Integration Test, Boston Naming Test, Animal Naming Test, phonemic fluency tasks, and a 12-word list learning task	Age, sex, education, insurance status, crystallized intelligence, vascular risk factors (hypertension, diabetes, smoking, alcohol use, BMI), MRI markers (white matter hyperintensity volume, silent brain infarcts, cerebral volume)	Participants with no/light LTPA showed greater decline in processing speed compared with those performing moderate–heavy LTPA (β = −0.231 ± 0.112, *p* = 0.040). Decline in episodic memory was also greater (β = −0.223 ± 0.117, *p* = 0.057). Among cognitively unimpaired participants at baseline, episodic memory decline remained significant (β = −0.257 ± 0.116, *p* = 0.027). The magnitude of decline was comparable to approximately 10 years of cognitive aging.

Fraser et al. [30] found that in middle-aged adults, each additional 1 MET increase in total physical activity was associated with a 0.03% increase in hippocampal volume (*p* = 0.004), with an analogous but attenuating effect observed in older adults (0.05% increase per MET, *p* = 0.049). Effects were stronger among APOE-ε4 carriers (0.10% hippocampal volume increase per MET). Best et al. [19] demonstrated that greater maintenance of self-reported walking minutes per week over 10 years, reflecting a habitual physical activity behaviour and not gait speed, predicted significantly less hippocampal volume loss over the subsequent three years (*p* = 0.03), with individuals maintaining walking showing approximately 4.5% hippocampal volume loss compared with 6.2% in the average participant. Barha et al. [18] reported sex-specific effects, with maintenance of self-reported walking minutes per week over 10 years predicting larger left dorsolateral prefrontal cortex (DLPFC) volume in females (*p* < 0.01) and a differential association with left hippocampal volume by sex (females *p* = 0.03; males *p* = 0.06). Smith et al. [25] found a significant interaction between leisure-time physical activity and APOE-ε4 genotype on hippocampal volume change over 18 months (*p* = 0.024, partial η² = 0.054), with hippocampal volume decreasing by approximately 3% in the high-risk/low-physical activity group while remaining stable in the high-risk/high-physical activity group.

Regarding total and global grey matter volume, Arnardottir et al. [29] found that higher total accelerometer-measured physical activity counts per day were associated with greater baseline grey matter volume (β = 0.11, *p* = 0.044) and significantly less five-year grey matter decline (β = 0.14, *p* = 0.0037). Palta et al. [23] observed that self-reported midlife MVPA at middle levels was associated with greater cortical grey matter volume in late life (β = 0.14 SD, 95% CI 0.06–0.23). Region-specific findings beyond the hippocampus were also reported. Ai et al. [33] found that self-reported midlife MVPA was associated with greater surface area of the left middle frontal gyrus (DLPFC; cluster = 376.12 mm², cluster-wise *p* < 0.05). Hotz et al. [32] reported that a higher baseline physical leisure activity score predicted less thinning of the right entorhinal cortex over seven years (β = 0.24, *p* < 0.05), with a thicker baseline entorhinal cortex independently predicting less memory decline. Huang et al. [34] found that higher self-reported leisure-time physical activity was associated with reduced dementia risk (HR = 0.78, 95% CI 0.70–0.93) and with greater volumes in the hippocampus, orbitofrontal cortex, middle temporal gyrus, and thalamus. Best et al. [20] reported that greater maintenance of self-reported walking minutes per week over 10 years was associated with greater middle frontal gyrus and anterior cingulate cortex volume and lower white matter hyperintensity burden, though no association was found with hippocampal volume in this conference abstract.

#### White matter

Six studies examined associations between physical activity and white matter integrity, assessed via DTI metrics (FA, MD, AD), white matter volume, or white matter hyperintensity (WMH) burden (Table 5).

Tian et al. [26] reported that each additional 22 min per day of accelerometer-measured relative MVPA was associated with slower decline in FA of the uncinate fasciculus (β = 0.035, *p* = 0.005) and hippocampal cingulum (β = 0.034, *p* = 0.01), as well as slower increase in MD of the parahippocampal gyrus (β = −0.021, *p* = 0.01) and entorhinal cortex (β = −0.030, *p* = 0.01), over a mean follow-up of 3.8 years. No significant associations were observed with absolute MVPA. Palta et al. [23] found that higher midlife self-reported MVPA was associated with better white matter integrity in late life (FA difference: 0.13 SD, 95% CI 0.004–0.26; MD difference: −0.11 SD, 95% CI − 0.21 to − 0.004) and with reduced odds of lacunar infarcts (OR = 0.68; 95% CI 0.46–0.99).

Best et al. [19] demonstrated that greater maintenance of self-reported walking minutes per week over 10 years predicted smaller increases in grey matter MD and white matter AD (*p* < 0.01), and that changes in white matter AD were independently associated with cognitive changes (β = −0.20, *p* = 0.007). Arnardottir et al. [29] found that higher total accelerometer-measured physical activity counts per day were associated with less five-year white matter volume decline (β = 0.11, *p* = 0.030), and that higher sedentary time (defined as < 100 counts/min) was independently associated with greater five-year white matter atrophy (β = −0.11, *p* = 0.0007). Hotz et al. [32] found no direct association between white matter hyperintensities or lacunes and declarative memory decline over seven years, though physical leisure activity predicted preservation of entorhinal cortex thickness. Best et al. [20] reported that greater maintenance of self-reported walking minutes per week over 10 years was associated with smaller increases in grey matter MD, consistent with the findings of the 2017 [19] publication from the same cohort.

### Cognitive outcomes

#### Executive function

Executive function was assessed in five studies. The most commonly used tests included the Trail Making Test Parts A and B, the Stroop Test, the Digit Symbol Substitution Test (DSST), and verbal fluency tasks (Table 5).

James et al. [35] demonstrated a dose-response accumulation effect across adulthood, with a greater number of periods of self-reported leisure-time physical activity from age 36 to 69 years being associated with higher cognitive scores at age 69 (*p* < 0.01). Individuals who were physically active across all five assessed life periods had the strongest association with higher Addenbrooke’s Cognitive Examination-III (ACE-III) scores compared with those who were never active. Frederiksen et al. [37] found that self-reported physical activity (classified as active: >30 min activity ≥ 3 days/week) was associated with better executive function both at baseline (β = 0.51, 95% CI 0.13–0.90, *p* = 0.008) and at three-year follow-up (β = 0.24, 95% CI 0.10–0.38, *p* = 0.001) in older adults with age-related white matter changes. Barha et al. [18] reported that maintenance of self-reported walking minutes per week over 10 years predicted less decline in executive function and processing speed in females (DSST slope, *p* < 0.01), but not in males, suggesting sex-specific effects on executive function trajectories.

Whitaker et al. [27] demonstrated that in men, replacing 30 min per day of light physical activity with accelerometer-measured MVPA was associated with improved DSST performance (β = 0.07, 95% CI 0.01–0.14, *p* = 0.019). However, associations were largely non-significant in women. Gafni et al. [21] found that higher self-reported MVPA trajectories over 20 years were associated with better autonomic balance but were not significantly associated with most cognitive outcomes, including executive function, after multiple-comparison correction.

#### Processing speed

Processing speed was one of the most consistently examined cognitive domains across the included studies, assessed in three studies (Table 5). Frederiksen et al. [37] found significant associations between self-reported physical activity and processing speed at both baseline (β = 0.48, *p* = 0.005) and three-year follow-up (β = 0.15, *p* = 0.02). Greendale et al. [22] observed in midlife women that higher self-reported sport and exercise physical activity was associated with better concurrent Symbol Digit Modalities Test (SDMT) performance (β = 0.36, 95% CI 0.14–0.59, *p* = 0.002) and with a smaller decline in processing speed over time (β = 0.06/year, *p* = 0.001); however, these associations were attenuated and became non-significant after full adjustment for demographic, menopause-related, and cardiometabolic covariates. Willey et al. [28] found that participants with no or light self-reported LTPA showed a significantly greater decline in processing speed than those performing moderate-to-heavy LTPA (β = −0.231, *p* = 0.040), with the magnitude of decline equivalent to approximately 10 years of cognitive aging.

#### Episodic memory

Episodic memory was assessed in four studies, with the Rey Auditory Verbal Learning Test (RAVLT) and various word-learning and recall paradigms being the most commonly used instruments (Table 5). Reas et al. [24] found that self-reported physical activity at concurrent assessments was associated with cognitive performance equivalent to that of individuals 1–2 years younger at age 70 and approximately 2–2.5 years younger at age 90. Participants who were active at both age 30 and older age maintained the highest global cognitive function with advancing age (*p* = 0.002). Willey et al. [28] observed greater episodic memory decline in participants with no or light LTPA, with the effect remaining significant after excluding individuals with baseline cognitive impairment (β = −0.257, *p* = 0.027). Whitaker et al. [27] demonstrated that in men, replacing sedentary time with accelerometer-measured MVPA was associated with improved RAVLT performance (β = 0.09, 95% CI 0.02–0.17, *p* = 0.009), while replacing light physical activity with sedentary time was associated with worse RAVLT scores (β = −0.03, 95% CI − 0.05 to − 0.01, *p* = 0.014). Hotz et al. [32] found that a higher physical leisure activity score at baseline predicted less thinning of the right entorhinal cortex, which in turn was independently associated with less decline in declarative memory performance (β = 0.54, *p* < 0.05).

#### Global cognition and accumulation effects across the lifespan

Several studies examined overall global cognition or cumulative effects of physical activity across multiple life stages (Table 5). Barha et al. [18] reported that maintaining self-reported walking minutes per week over 10 years was associated with less decline in global cognition, as measured by the Modified Mini-Mental State Examination, in both males and females (*p* = 0.03). Best et al. [19] found that greater maintenance of self-reported walking minutes per week was associated with better maintenance of global cognitive performance over 3 years (*p* < 0.01). James et al. [35] provided compelling dose-response evidence that each additional period of self-reported leisure-time physical activity across adulthood was independently associated with higher ACE-III scores at age 69, even after adjustment for childhood cognition, socioeconomic position, and cardiovascular health. Gafni et al. [21] examined 20-year self-reported MVPA trajectories in young adults and found that higher trajectories were associated with better autonomic balance (higher heart rate variability, *p* < 0.001; lower resting heart rate, *p* < 0.0001) and with category fluency mediated via heart rate variability, though most cognitive associations did not survive multiple comparison correction.

Ai et al. [33] examined longitudinal cognitive change over four years using the Repeatable Battery for the Assessment of Neuropsychological Status (RBANS), which assessed immediate memory, visuospatial ability, language, attention, and delayed memory. Despite observing significant associations between self-reported midlife MVPA and greater left middle frontal gyrus surface area, no significant association was found between midlife physical activity and longitudinal change in any RBANS cognitive domain over the follow-up period. Similarly, Smith et al. [25] assessed global cognition using the Mini-Mental State Examination (MMSE) and Mattis Dementia Rating Scale-2 (DRS-2), along with functional status and depressive symptoms, over 18 months. No significant associations were observed between self-reported leisure-time physical activity and cognitive outcomes, despite the significant interaction observed between physical activity and APOE-ε4 status on hippocampal volume change.

### Bidirectional associations

Two studies investigated the directionality of the physical activity–brain structure relationship using bidirectional analytical frameworks (Table 5). Hofman et al. [31] found that larger total brain volume (β = 0.067, pFDR = 0.001), grey matter volume (β = 0.063, pFDR = 0.002), and white matter volume (β = 0.051, pFDR = 0.013) at baseline predicted higher self-reported sports participation at follow-up, while baseline physical activity did not significantly predict subsequent brain structural changes. Rodriguez-Ayllon et al. [36] found that greater baseline hippocampal volume (β = 0.048, pFDR = 0.016) and global fractional anisotropy (β = 0.042, pFDR = 0.028) predicted higher self-reported household physical activity at follow-up, though self-reported walking at baseline was negatively associated with white matter volume change over time (β = −0.026, pFDR = 0.008).

### Risk of bias in included studies

**Fig. 2:**
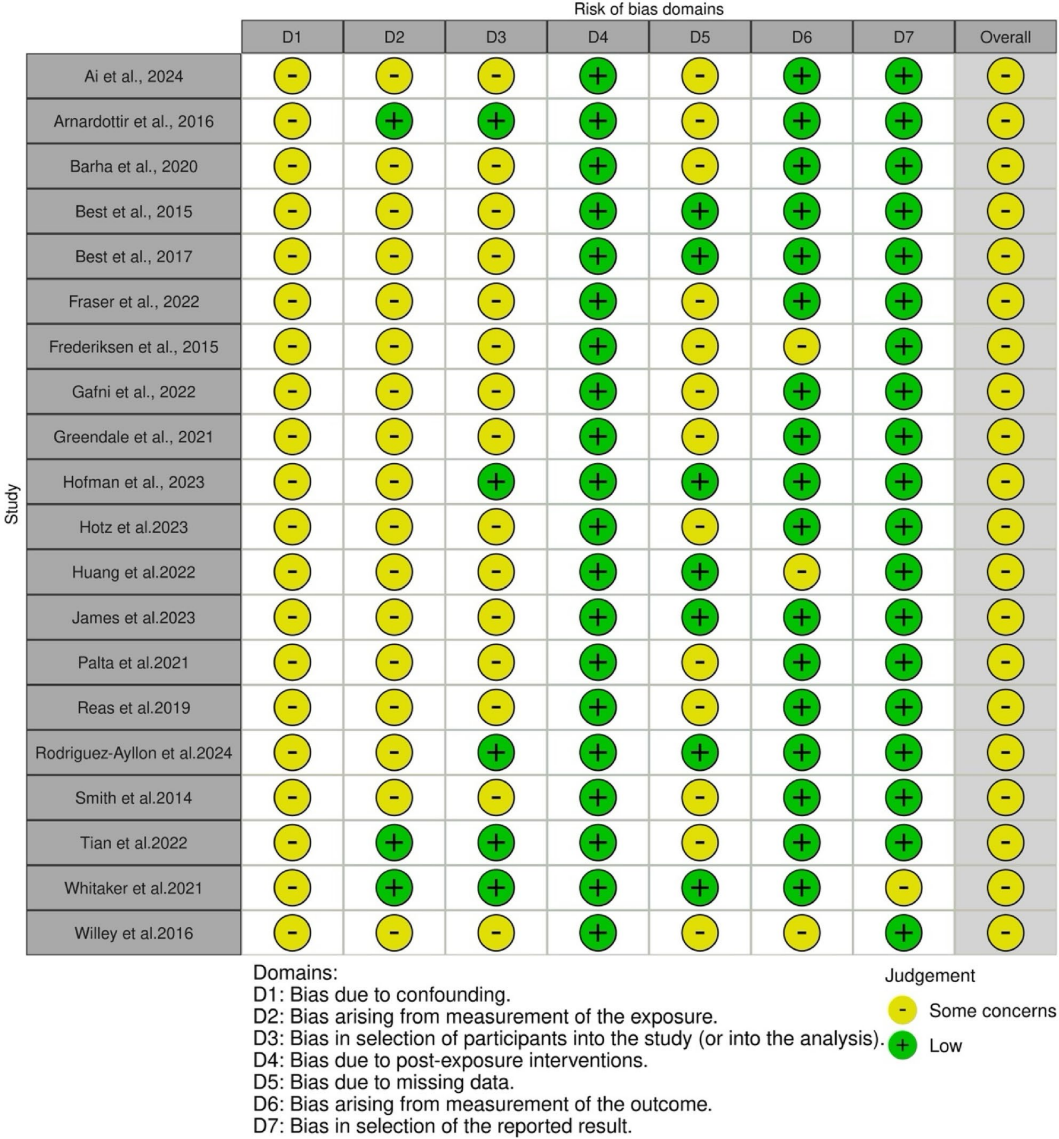
Risk of bias assessment of the included studies

The risk of bias of all 28 included studies was assessed using the ROBINS-E tool across seven domains. The traffic light plot was generated using the RobVis visualization tool [38]. All 20 studies received an overall judgment of “some concerns,” with no study rated as low risk of bias across all domains. The traffic light plot is presented in Fig. 2.

#### Domain 1

Bias due to confounding was rated as “some concerns” in all 20 included studies, representing the most pervasive source of bias across this review. This was anticipated given the observational nature of the included designs, in which physical activity exposure is self-selected rather than randomly assigned. Confounding arises when unmeasured or inadequately controlled variables, such as baseline health status, socioeconomic position, education, diet, sleep quality, and comorbid cardiometabolic conditions, are independently associated with both physical activity engagement and brain structural outcomes. Although most included studies adjusted for age, sex, and education, few accounted for all plausible confounders. The direction of this bias is most likely towards overestimating the true association between physical activity and brain outcomes, as physically active individuals tend to have more favorable overall health profiles. Readers should therefore interpret the reported associations as reflecting the totality of health behaviours and characteristics that co-occur with physical activity, rather than the isolated effect of physical activity alone.

#### Domain 2

Bias arising from exposure measurement was rated as “some concerns” in seveteen studies [18–25, 28, 30–37]. These studies with concerns relied on self-reported physical activity instruments. Self-report measures are subject to social desirability bias, recall error, and imprecision in capturing intensity and duration, all of which introduce non-differential measurement error that typically attenuates true effect sizes towards the null.

#### Domain 3

Bias in selection of participants into the study or analysis was rated as low risk in five studies [26, 27, 29, 31, 36] and as “some concerns” in the remaining 15. The primary concern in most studies was selective attrition, in which participants lost to follow-up differed systematically from those who remained in the analysis. In studies of ageing populations, those who are less healthy, less physically active, or experiencing early cognitive decline are disproportionately likely to withdraw or die before neuroimaging follow-up. This healthy survivor bias means that participants who contribute complete data to the analyses are systematically healthier than the broader target population, likely leading to underestimation of the protective association between physical activity and brain outcomes, or to an overly optimistic characterization of the trajectory of brain structural change in physically active individuals.

#### Domain 4

Bias due to post-exposure interventions was rated as low risk in all the included studies. This reflects the observational nature of the designs, in which no structured co-intervention was introduced between exposure assessment and outcome measurement that could plausibly confound the observed associations.

#### Domain 5

Bias due to missing data was rated as low risk in seven studies [19, 20, 27, 31, 34–36] and as “some concerns” in the remaining 13. Studies with concerns either did not report the extent of missing data, used complete-case analysis without sensitivity testing, or did not apply multiple imputation or other principled methods for handling missing observations. The direction of this bias is again likely to be towards underestimating protective effects, for the same healthy-survivor reasons described under Domain 3.

#### Domain 6

Bias arising from the measurement of the outcome was rated as “some concerns” in three studies [28, 34, 37] and as low risk in the remaining 17 studies. The concern in these three studies related primarily to the use of cognitive assessment instruments that were administered without blinding to physical activity status, or to the reliance on administrative or self-reported cognitive or dementia outcome data that may be subject to ascertainment or classification errors. In studies using standardized neuroimaging protocols with automated volumetric or diffusion tensor imaging analysis pipelines, the majority of included studies had a low risk of outcome measurement bias, as automated analytical methods are not susceptible to assessor knowledge of exposure status.

#### Domain 7

Bias in the selection of the reported result was rated as “some concerns” in one study [27] and as low risk in the remaining 19 studies. The concern in Whitaker et al. [27] related to the multiplicity of analyses reported across a large number of physical activity and cognitive outcome combinations, without a pre-specified primary analysis or correction for multiple comparisons, raising the possibility of selective emphasis on statistically significant findings.

Taken together, the dominant sources of bias in this review are confounding, selection bias due to healthy survivor attrition, and non-differential exposure measurement error due to self-report. These biases collectively suggest that the true associations between long-term physical activity and brain structural and cognitive outcomes may be both overestimated in magnitude and underestimated in precision.

## Discussion

This systematic review synthesized longitudinal observational evidence from 20 studies to examine associations between long-term habitual physical activity and brain structure and cognitive function across different adult age groups. The principal observations of this review are threefold. First, higher levels of habitual physical activity were associated with preserved grey matter volume and with attenuated white matter microstructural decline. Second, long-term physical activity was associated with better performance across multiple cognitive domains, including executive function, processing speed, and episodic memory, with some evidence of a dose-response accumulation pattern across the adult lifespan. Third, the relationship between brain structure and physical activity may not be unidirectional, with preserved brain structural integrity also appearing to predict subsequent physical activity engagement.

### Brain structural findings

Multiple studies in this review observed an association between higher habitual physical activity and greater hippocampal volume or slower hippocampal atrophy, a finding consistent with the broader neuroimaging literature. These observations are biologically plausible given the well-documented role of aerobic activity in upregulating trophic factors, including BDNF and IGF-1, and in supporting cerebrovascular perfusion, both of which are thought to sustain hippocampal neuroplasticity [38]. The experimental evidence of Erickson et al. [5] demonstrated that aerobic exercise training was associated with increased anterior hippocampal volume in an RCT; the present review adds longitudinal observational evidence that associations between sustained habitual walking, leisure-time physical activity, and preserved hippocampal volume are detectable across follow-up periods ranging from 18 months to 12 years in free-living populations. Fraser et al. [29] observed a graded association between total MET-based physical activity and hippocampal volume, and reported stronger associations among APOE-ε4 carriers; this pattern was also observed by Smith et al. [24], who found that lower physical activity was associated with greater hippocampal volume loss specifically in APOE-ε4 carriers over 18 months. Taken together, these observations are consistent with the hypothesis that habitual physical activity may be associated with reduced hippocampal atrophy, with a potentially amplified association in genetically at-risk individuals.

Across several studies, associations were also observed between higher physical activity and preservation of prefrontal grey matter volume, including the dorsolateral prefrontal cortex and middle frontal gyrus. These observations are plausible given the prefrontal cortex’s vulnerability to early age-related atrophy and its dependence on intact cerebrovascular supply [39]. Ai et al. [32] observed that higher midlife self-reported MVPA was associated with greater left middle frontal gyrus surface area, while Barha et al. [17] found associations between walking maintenance and DLPFC volume.

Regarding white matter integrity, Tian et al. [25] observed that higher accelerometer-measured relative MVPA was associated with slower decline in FA of the uncinate fasciculus and hippocampal cingulum, tracts implicated in episodic memory and temporal lobe connectivity [40]. Palta et al. [22] found associations between midlife MVPA and better late-life white matter integrity alongside reduced odds of lacunar infarcts, consistent with a potential cerebrovascular pathway. Arnardottir et al. [28] observed that higher sedentary time was independently associated with greater white matter atrophy, suggesting that sedentary behaviour may be an independently relevant exposure target distinct from physical activity per se. These associations are consistent with mechanistic evidence suggesting that aerobic activity may support cerebrovascular health, reduce arterial stiffness, and attenuate neuroinflammation [41].

### Cognitive findings

The observed associations between long-term physical activity and cognitive outcomes in this review are broadly consistent with prior meta-analytic evidence linking physical activity to executive function, processing speed, and memory in older adults [42]. The present review adds to that literature by documenting that these associations are detectable in longitudinal observational settings across follow-up durations of up to 30 years, and that the pattern of associations across studies suggests a possible dose-response accumulation effect across the life course. James et al. [34] observed that each additional period of self-reported leisure-time physical activity across five assessments from age 36 to 69 years was associated with higher ACE-III scores at age 69, after adjustment for childhood cognition, socioeconomic position, and cardiovascular health. This pattern is consistent with a life-course model of physical activity and cognitive reserve accumulation [43].

Reas et al. [23] found that physically active participants showed cognitive performance consistent with approximately 2 years of cognitive age at age 90, providing a clinically interpretable estimate of the magnitude of the observed association. Processing speed emerged as one of the most consistently associated cognitive domains across the included studies. Willey et al. [27] observed that participants reporting no or light leisure-time physical activity showed greater processing speed decline, with the magnitude equivalent to approximately 10 additional years of cognitive aging.

Importantly, not all studies observed robust cognitive associations. Greendale et al. [21] found that associations between physical activity and processing speed in midlife women were attenuated to non-significance after adjustment for menopause-related variables, hypertension, and diabetes, suggesting that cardiometabolic health may mediate or confound the observed association. Gafni et al. [20] found that most associations between 20-year MVPA trajectories and cognitive outcomes did not survive multiple comparison correction. Ai et al. [32] observed no significant association between midlife MVPA and longitudinal cognitive change over four years, despite observing significant brain structural associations. These null findings are important and should not be dismissed; they suggest that structural brain changes associated with physical activity may not necessarily translate into detectable cognitive benefits over the follow-up periods examined, and that the brain-cognition relationship may itself be moderated by age, baseline cognitive reserve, and measurement sensitivity.

Across the 20 included studies, executive function and processing speed showed the most consistent directional associations with physical activity, while episodic memory associations were observed in fewer studies and with greater variability. Global cognitive outcomes showed mixed findings: some studies reported significant associations, while others reported null results after covariate adjustment. This pattern suggests that the associations may be domain-specific rather than reflecting global cognitive enhancement.

### Moderating factors

Several included studies identified sex as a potential moderator of associations between physical activity, brain structure, and cognitive outcomes. Barha et al. [17] observed that walking maintenance was associated with better executive function, processing speed, and DLPFC volume in females but not males, while Whitaker et al. [26] found that cognitive associations with replacing sedentary behaviour with MVPA were significant in men but not women. These divergent directional patterns preclude a definitive conclusion about sex moderation at this stage. Plausible mechanisms include estrogen-mediated regulation of BDNF expression, sex differences in cerebrovascular risk profiles, and differential patterns of age-related brain atrophy between males and females [44].

Regarding genetic moderation, Smith et al. [24] and Fraser et al. [29] both observed stronger associations between physical activity and hippocampal outcomes in APOE-ε4 carriers than in non-carriers. These observations are consistent with the hypothesis that physical activity may partially offset the neurobiological consequences of the APOE-ε4 allele, including impaired amyloid clearance, reduced cerebrovascular reactivity, and diminished synaptic plasticity [45].

### Bidirectionality

Two studies employed bidirectional analytical frameworks. Hofman et al. [30] and Rodriguez-Ayllon et al. [35] found that baseline brain structural measures, including total brain volume, grey matter volume, hippocampal volume, and global fractional anisotropy, were associated with subsequent physical activity levels, while baseline physical activity did not significantly predict subsequent brain structural change. This pattern is consistent with a reverse causation model, in which preserved brain integrity enables continued physical activity engagement in older age, rather than physical activity preserving brain structure.

### Age-Specific Considerations

The included studies were predominantly conducted in older adult populations, with relatively few examining middle-aged or younger adults. Among studies in younger and middle-aged cohorts, Gafni et al. [20] and James et al. [34] observed that physical activity patterns in early adulthood were associated with cognitive outcomes decades later, consistent with a life-course perspective on brain health that is not confined to late life. The predominance of older adult studies in the existing literature represents a gap, as the period of greatest potential for accumulating physical activity-related neurobiological reserves may be in mid-life, when atrophy trajectories are beginning, but structural brain changes are still potentially modifiable.

### Limitations of the studies included in the review

Several methodological considerations limit the strength of conclusions that can be drawn from this review. First, all included studies were observational, and none can establish causal relationships between physical activity and brain outcomes. Residual confounding from unmeasured variables remains a plausible alternative explanation for observed associations in all included studies. Second, the near-universal reliance on self-reported physical activity measures introduces non-differential measurement error that likely attenuates true effect sizes. Accelerometry was employed in only three of the 20 included studies. Third, the heterogeneity of neuroimaging acquisition protocols, MRI field strengths, and analytical pipelines across studies limits direct comparability of findings and precludes quantitative pooling of effect estimates. Fourth, the predominance of studies conducted in high-income, predominantly White Western populations limits the generalizability of findings to other racial, ethnic, and socioeconomic groups.

### Limitations of the current review

It is important to consider how this heterogeneity affects the interpretation of the synthesized evidence. Heterogeneity in physical activity measurement is particularly consequential. Studies using accelerometry capture objective movement data with greater precision than self-report instruments, which are subject to recall bias, social desirability effects, and imprecision in intensity quantification. Non-differential measurement error from self-report typically attenuates associations towards the null, meaning that true associations may be somewhat stronger than observed in self-report studies, though this cannot be confirmed without head-to-head comparisons within the same cohorts. Heterogeneity in neuroimaging protocols limits the ability to draw quantitative conclusions about the magnitude of associations, even where directional consistency is observed. Heterogeneity in follow-up duration varies considerably in their capacity to detect brain structural changes, which unfold over years to decades.

These sources of heterogeneity collectively mean that the narrative synthesis presented in this review reflects a pattern of directionally consistent but quantitatively heterogeneous associations, rather than a precise estimate of the effect of physical activity on brain outcomes.

#### Future recommendations

Future longitudinal studies should prioritize objective physical activity measurement using validated accelerometry protocols, standardize neuroimaging acquisition and processing pipelines, and routinely collect data on potential moderating variables, including sex, APOE genotype, hormonal status, and cardiometabolic comorbidity. Longer follow-up periods spanning multiple decades are needed to characterize the full trajectory of brain structural and cognitive change in relation to evolving physical activity patterns. Studies employing causal inference methods, including Mendelian randomization, time-varying marginal structural models, and natural experiments exploiting policy-level changes in physical activity opportunities, are particularly needed to address the fundamental limitation of reverse causation in this field.

## Conclusion

This systematic review synthesized longitudinal observational evidence from the studies examining associations between long-term habitual physical activity, brain structure, and cognitive function across the adult lifespan. Across the included studies, higher levels of habitual physical activity were consistently associated with preserved hippocampal and prefrontal grey matter volume, attenuated white matter microstructural decline, and better performance across executive function, processing speed, and episodic memory domains. It must be emphasized that all included studies were observational, and the associations reported in this review cannot be interpreted as evidence of causation. Notwithstanding these limitations, the directional consistency of associations across methodologically heterogeneous studies is consistent with the hypothesis that sustained habitual physical activity engagement across adulthood may contribute to the preservation of brain structure and attenuation of cognitive decline. These findings support the continued prioritization of physical activity promotion within public health and dementia prevention strategies, while highlighting the urgent need for future research that employs objective exposure assessment, standardized neuroimaging protocols, and causal inference methods to address the fundamental limitations of observational designs in this domain.

## Supplementary Information

Supplementary Material 1.

## Data Availability

The data that support the findings of this study are openly available in the public domain.
