# Supplementary material for: Long-term physical activity, brain structure, and cognitive function: a systematic review of longitudinal observational evidence
Source: BMC Neurol. 2026 Jun 11;26:522. doi: 10.1186/s12883-026-05055-5 (PMC13483407; doi:10.1186/s12883-026-05055-5)
Supplement: Supplementary file 1 [file 12883_2026_5055_MOESM1_ESM.docx]

**Pubmed**

**("physical activity" OR exercise OR "physical fitness" OR "aerobic exercise" OR walking OR running OR jogging OR "cardiorespiratory fitness" OR CRF OR "sedentary" OR "sedentary behaviour" OR "sedentary behavior" OR "sedentary lifestyle" OR "resistance training" OR "resistance exercise") AND ("brain volume" OR "grey matter" OR "gray matter" OR "white matter" OR "hippocampal volume" OR hippocampus OR "prefrontal cortex" OR "cortical thickness" OR "brain structure" OR "neuroimaging" OR "magnetic resonance imaging" OR MRI OR "fractional anisotropy" OR "mean diffusivity" OR "white matter integrity" OR "axial diffusivity" OR "radial diffusivity" OR "neuroplasticity") AND (cognition OR "cognitive function" OR "cognitive performance" OR memory OR attention OR "executive function" OR "processing speed") AND (adult OR "young adult" OR "middle aged adult" OR "older adult" OR elder OR aged)** Filters: **Full text, English, Humans, Adult: 19+ years, Young Adult: 19-24 years, Adult: 19-44 years, Middle Aged + Aged: 45+ years, Middle Aged: 45-64 years, Aged: 65+ years, 80 and over: 80+ years**

**Scopus**

( TITLE-ABS-KEY ( "physical activity" OR exercise OR "physical fitness" OR "aerobic exercise" OR walking OR running OR jogging OR "cardiorespiratory fitness" OR CRF OR "sedentary" OR "sedentary behaviour" OR "sedentary behavior" OR "sedentary lifestyle" OR "resistance training" OR "resistance exercise" ) AND TITLE-ABS-KEY ( "brain volume" OR "grey matter" OR "gray matter" OR "white matter" OR "hippocampal volume" OR hippocampus OR "prefrontal cortex" OR "cortical thickness" OR "brain structure" OR "neuroimaging" OR "magnetic resonance imaging" OR MRI OR "fractional anisotropy" OR "mean diffusivity" OR "white matter integrity" OR "axial diffusivity" OR "radial diffusivity" OR "neuroplasticity" ) AND TITLE-ABS-KEY ( cognition OR "cognitive function" OR "cognitive performance" OR memory OR attention OR "executive function" OR "processing speed" ) AND TITLE-ABS-KEY ( adult OR "young adult" OR "middle aged adult" OR "older adult" OR elder OR aged ) ) AND ( LIMIT-TO ( PUBYEAR , "2020" ) OR LIMIT-TO ( PUBYEAR , "2021" ) OR LIMIT-TO ( PUBYEAR , "2022" ) OR LIMIT-TO ( PUBYEAR , "2023" ) OR LIMIT-TO ( PUBYEAR , "2024" ) ) AND ( LIMIT-TO ( DOCTYPE , "ar" ) ) AND ( LIMIT-TO ( LANGUAGE , "English" ) ) AND ( LIMIT-TO ( PUBSTAGE , "final" ) ) AND ( LIMIT-TO ( SRCTYPE , "j" ) )

**Embase**

('physical activity' OR exercise OR 'physical fitness' OR 'aerobic exercise' OR walking OR running OR jogging OR 'cardiorespiratory fitness' OR crf OR 'sedentary' OR 'sedentary behaviour' OR 'sedentary behavior' OR 'sedentary lifestyle' OR 'resistance training' OR 'resistance exercise') AND ('brain volume' OR 'grey matter' OR 'gray matter' OR 'white matter' OR 'hippocampal volume' OR hippocampus OR 'prefrontal cortex' OR 'cortical thickness' OR 'brain structure' OR 'neuroimaging' OR 'magnetic resonance imaging' OR mri OR 'fractional anisotropy' OR 'mean diffusivity' OR 'white matter integrity' OR 'axial diffusivity' OR 'radial diffusivity' OR 'neuroplasticity') AND (cognition OR 'cognitive function' OR 'cognitive performance' OR memory OR attention OR 'executive function' OR 'processing speed') AND (adult OR 'young adult' OR 'middle-aged adult' OR 'older adult' OR elder OR aged) AND [<1966-2024]/py AND 'article'/it

**Web of science**

<https://www.webofscience.com/wos/woscc/summary/9eff17fa-0851-485b-a835-4cf7f39e6b88-01b34ec2f6/relevance/1>

Ovid

(("physical activity" or exercise or "physical fitness" or "aerobic exercise" or walking or running or jogging or "cardiorespiratory fitness" or CRF or "sedentary" or "sedentary behaviour" or "sedentary behavior" or "sedentary lifestyle" or "resistance training" or "resistance exercise") and ("brain volume" or "grey matter" or "gray matter" or "white matter" or "hippocampal volume" or hippocampus or "prefrontal cortex" or "cortical thickness" or "brain structure" or "neuroimaging" or "magnetic resonance imaging" or MRI or "fractional anisotropy" or "mean diffusivity" or "white matter integrity" or "axial diffusivity" or "radial diffusivity" or "neuroplasticity") and (cognition or "cognitive function" or "cognitive performance" or memory or attention or "executive function" or "processing speed") and (adult or "young adult" or "middle-aged adult" or "older adult" or elder or aged)).af.

limit 1 to yr="1861 - 2024"

limit 2 to english language
